# Sex differences in the prognosis of patients with hypertrophic cardiomyopathy

**DOI:** 10.1038/s41598-021-84335-1

**Published:** 2021-03-01

**Authors:** Minkwan Kim, Bongsung Kim, You-Jung Choi, Hyun-Jung Lee, Heesun Lee, Jun-Bean Park, Seung-Pyo Lee, Kyung-Do Han, Yong-Jin Kim, Hyung-Kwan Kim

**Affiliations:** 1 Department of Internal Medicine and Cardiovascular Center, Seoul National University Hospital, Seoul, Republic of Korea; 2 Division of Cardiology, Department of Internal Medicine, Yongin Severance Hospital, Yonsei University College of Medicine, Yongin-si, Gyeonggi-do Republic of Korea; 3 Department of Statistics and Actuarial Science, The Soongsil University, Seoul, Republic of Korea; 4 Division of Cardiology, Department of Internal Medicine, Seoul National University Hospital Healthcare System Gangnam Center, Seoul, Republic of Korea

**Keywords:** Cardiology, Cardiomyopathies

## Abstract

We investigated sex-related differences in the prognosis of patients with hypertrophic cardiomyopathy (HCM) using the Korea National Health Insurance Service database. From 2010 to 2016, 9524 patients diagnosed with HCM and had more than 1-year follow-up period were analyzed. The primary endpoint was the composite of cardiovascular death or new-onset heart failure (HF) admission. Propensity score-matching analysis was performed to adjust for different baseline characteristics. With a 4.4-years’ median follow-up interval (range 2.0–6.6 years) and male predominance (77.6%), women with HCM were older (52.6 ± 9.7 vs. 51.4 ± 9.1, *p* < 0.001), had lower incomes, more comorbidities based on Charlson comorbidity index. Women with HCM had a higher incidence of the primary endpoint than men (incidence rate: 34.15 vs. 22.83 per 1000 person-years, log-rank *p* < 0.001). Multivariable Cox analysis showed that female sex was a poor prognostic factor for the primary endpoint (HR 1.43, 95% CI 1.24–1.64, *p* < 0.001). This was mainly driven by a higher incidence of new-onset HF admission (HR 1.55, 95% CI 1.34–1.80). However, there was no difference in the incidence of cardiovascular death between the sexes. This result was concordant in the propensity score-matched cohort. In conclusion, women with HCM have worse prognosis, which was mainly driven by a higher new-onset HF admission.

## Introduction

Hypertrophic cardiomyopathy (HCM) is a genetic cardiac disease that is transmitted in an autosomal dominant fashion with extreme diversity in clinical presentation and natural history^1,2^. Despite an autosomal dominant transmission, earlier investigations of the disease frequently reported male predominance in prevalence^3–7^, suggesting sex differences in clinical expression. Although prognostic differences according to sex in a variety of acquired cardiac diseases including coronary artery disease, atrial fibrillation, valvular heart disease, and aortic diseases have been reported^8–12^, there have been conflicting results regarding sex differences in the prognosis of HCM^6,7,13–15^. Given the improved prognosis of HCM patients in the contemporary management era, a larger number of HCM patients may be required to draw more solid conclusion. Differences in the baseline characteristics, especially older age in women than men, also made it difficult to draw a definite conclusion^3,5–7,16^. Therefore, this study was designed to investigate sex differences in the prognosis of HCM by using a large database of the Korea National Health Insurance Service (NHIS). In addition, we utilized the propensity score (PS)-matching analysis to balance the differences in the baseline characteristics of both sexes.

## Results

### Baseline characteristics of the study subjects

A total of 9524 patients were finally included in this study (mean age 51.7 ± 9.3 years); the study population was predominantly male (77.6%, n = 7388). The baseline clinical characteristics of the patients are summarized in Table 1. Generally, Women were one year older than men (52.6 ± 9.7 vs. 51.4 ± 9.1, *p* < 0.001) and had a higher Charlson comorbidity index (CCI) (2.31 ± 1.98 vs. 1.95 ± 1.89, *p* < 0.001), but were less likely to have a history of chronic kidney disease, ischemic heart disease (IHD) requiring coronary intervention, and myocardial infarction.

**Table 1:** Baseline characteristics in the original cohort of patients with HCM.

	Total (N = 9524)	Men (N = 7388)	Women (N = 2136)	*p*
Age	51.7 ± 9.3	51.4 ± 9.1	52.6 ± 9.7	< 0.001
Income, low 20%	1616 (17.0)	1133 (15.3)	483 (22.6)	< 0.001
**Underlying disease, n (%)**				
Hypertension	3956 (41.5)	3089 (41.8)	867 (40.6)	0.313
Diabetes mellitus	890 (9.3)	703 (9.5)	187 (8.8)	0.287
Dyslipidemia	2346 (24.6)	1819 (24.6)	527 (24.7)	0.961
IHD requiring coronary intervention	94 (1.0)	82 (1.1)	12 (0.6)	0.024
Myocardial infarction	243 (2.6)	203 (2.8)	40 (1.9)	0.024
Atrial fibrillation	612 (6.4)	479 (6.5)	133 (6.2)	0.670
Previous stroke	420 (4.4)	323 (4.4)	97 (4.5)	0.737
Chronic kidney disease	234 (2.5)	195 (2.6)	39 (1.8)	0.032
End-stage renal disease	63 (0.7)	51 (0.7)	12 (0.6)	0.519
**Medication, n (%)**				
RAS blocker	2934 (30.8)	2307 (31.2)	627 (29.4)	0.099
Beta-blocker	3387 (35.6)	2628 (35.6)	759 (35.5)	0.975
Calcium channel blocker	1393 (14.6)	1087 (14.7)	306 (14.3)	0.656
Antiplatelet agent	2783 (29.2)	2188 (29.6)	595 (27.9)	0.115
Statin	2334 (24.5)	1803 (24.4)	531 (24.9)	0.667
Charlson comorbidity index	2.03 ± 1.92	1.95 ± 1.89	2.31 ± 1.98	< 0.001

*HCM* hypertrophic cardiomyopathy, *IHD* ischemic heart disease, *RAS* renin–angiotensin system.

### Clinical outcomes in the original cohort

The median follow-up duration was 4.4 (interquartile range [IQR] 2.0–6.6) years or 39,267 patient-years (4.4 [IQR 1.9–6.1] years for women; 4.5 [IQR 2.1–6.7] years for men). During follow-up, the primary endpoint was reached in 956 (10.0%) patients; cardiovascular death in 227 (2.4%) patients, and new-onset heart failure (HF) admission in 804 (8.4%) patients. Seventy-five patients experienced both new-onset HF admission and cardiovascular death events, for whom the first event, i.e., new-onset HF admission, was included in the primary endpoint analysis. All-cause death was observed in 379 patients (4.0%).

**Figure 1:**
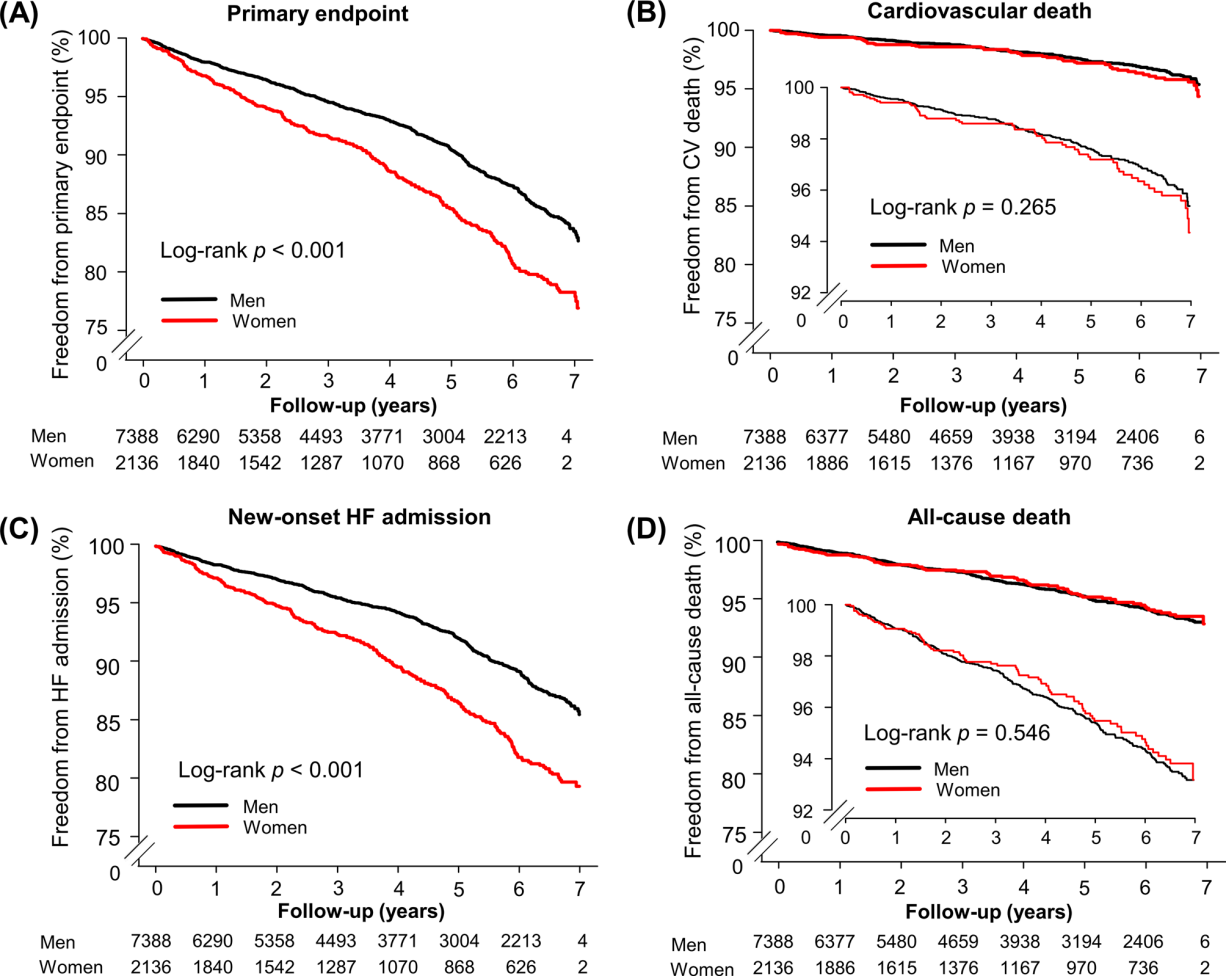
Kaplan–Meier curve of the cumulative incidence of clinical events in the original cohort of patients with hypertrophic cardiomyopathy. (**A**) The primary endpoint of cardiovascular death or new-onset HF admission; (**B**) cardiovascular death; (**C**) new-onset HF admission; (**D**) all-cause death. *CV* cardiovascular, *HF* heart failure.

According to the Kaplan–Meier survival curves (Fig. 1), women had a higher incidence of new-onset HF admission than men (*p* < 0.001), whereas the incidences of cardiovascular death in women and men were similar (*p* = 0.265). Therefore, the significantly higher occurrence of the primary endpoint in women was mainly driven by a greater number of new-onset HF admission (34.15 vs. 22.83 per 1000 person-years for women and men, respectively, *p* < 0.001) (Table 2). There was no difference between the sexes regarding all-cause death (*p* = 0.546) (Fig. 1D).

**Table 2:** Clinical outcome in the original cohort of patients with HCM.

	Numbers	Events	Incidence rate (per 1000)	*p*
**Primary endpoint**	9524	956	25.36	< 0.001
Men	7388	668	22.83	
Women	2136	288	34.15	
**Cardiovascular death**	9524	227	5.78	0.265
Men	7388	167	5.52	
Women	2136	60	6.67	
**New-onset HF admission**	9524	804	21.33	< 0.001
Men	7388	547	18.69	
Women	2136	257	30.47	
**All-cause death**	9524	379	9.65	0.546
Men	7388	297	9.81	
Women	2136	82	9.12	

*HCM* hypertrophic cardiomyopathy, *HF* heart failure.

Univariate Cox analysis revealed significant differences in baseline characteristics between men and women, including sex, age, CCI, income, hypertension, dyslipidemia, chronic kidney disease, IHD requiring coronary intervention, atrial fibrillation, and use of beta-blocker (Supplementary Table 1). Multivariate Cox regression analysis conducted after adjusting for the clinical differences in the univariate Cox analysis showed that the female sex remained a significant poor prognosticator for predicting the primary endpoint in two different multivariable models of the original cohort. Of note, this finding was mainly driven by the significantly higher incidence of new-onset HF admission among women with HCM, and there was no statistical difference in the incidences of cardiovascular death or all-cause death in two different multivariable models (Table 3).

**Table 3:** Sex differences in the multivariable Cox regression analysis in the original and the propensity score-matched cohorts of patients with HCM.

Variables	Unadjusted		Model 1^a^		Model 2^b^		Propensity score-matched	
	HR for women (95% CI)	*p*	HR for women (95% CI)	*p*	HR for women (95% CI)	*p*	HR for women (95% CI)	*p*
Primary endpoint	1.49 (1.30–1.71)	< 0.001	1.39 (1.21–1.60)	< 0.001	1.43 (1.24–1.64)	< 0.001	1.43 (1.22–1.68)	< 0.001
Cardiovascular death	1.18 (0.88–1.59)	0.266	1.07 (0.80–1.44)	0.645	1.11 (0.83–1.50)	0.478	1.27 (0.91–1.78)	0.162
New-onset HF admission	1.63 (1.41–1.89)	< 0.001	1.53 (1.31–1.77)	< 0.001	1.55 (1.34–1.80)	< 0.001	1.54 (1.30–1.82)	< 0.001
All-cause death	0.93 (0.73–1.18)	0.546	0.82 (0.64–1.05)	0.118	0.83 (0.65–1.07)	0.144	0.91 (0.69–1.21)	0.525

*HCM* hypertrophic cardiomyopathy, *HR* hazard ratio, *HF* heart failure.

^a^Multivariate clinical model 1 was adjusted for age and Charlson comorbidity index.

^b^Model 2 was adjusted for age, Charlson comorbidity index, low income, hypertension, dyslipidemia, ischemic heart disease requiring coronary intervention, chronic kidney disease, atrial fibrillation, and beta-blocker.

### Clinical outcomes in the propensity score-matched cohort

In the original HCM cohort, baseline clinical characteristics were significantly different between women and men (Table 1). Thus, to balance the baseline clinical characteristics of both sexes, we created a PS-matched HCM cohort. After PS-matching, each baseline clinical characteristic was well balanced (Supplementary Table 2).

**Figure 2:**
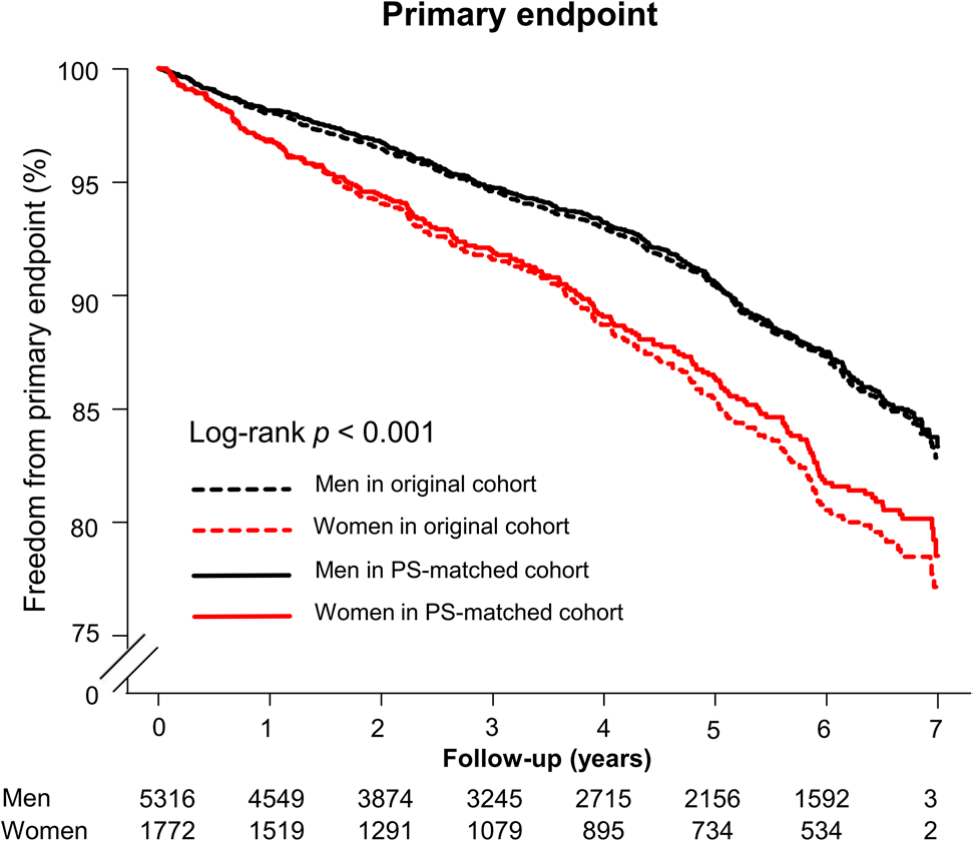
Kaplan–Meier curve of the cumulative incidence of the primary endpoint in the original and propensity score-matched cohort of patients with hypertrophic cardiomyopathy. The rate of occurrence of the primary endpoint, a composite of cardiovascular death or new-onset heart failure, was higher among women than among men, and this result was concordant with that in the propensity score-matched cohort. PS, propensity score.

In the PS-matched cohort, the rate of occurrence of the primary endpoint was also higher among women than among men (32.06 vs. 22.24 per 1,000 person-years, log-rank *p* < 0.001) (Fig. 2 and Supplementary Fig. 1A). The clinical outcomes of cardiovascular death, new-onset HF admission, and all-cause death were all consistent with those in the original cohort (Supplementary Fig. 1B–D). In the PS-matched cohort, multivariable Cox regression analysis showed that female sex remained a significant poor prognosticator for the primary endpoint (hazard ratio [HR] 1.43, 95% confidence interval [CI] 1.22–1.68; *p* < 0.001), a finding that was again driven by the higher incidence of new-onset HF admission among women (HR 1.54, 95% CI 1.30–1.82; *p* < 0.001) (Table 3).

### Sensitivity analysis of patients without hypertension

To eliminate the potential confounding effects of hypertension on increased ventricular wall thickness, we performed a sensitivity analysis for 5568 patients without the *International Classification of Disease, Tenth Revision* (ICD-10) code of hypertension in the original cohort. Baseline characteristics of the patients without hypertension were basically the same as those of the patients in the original cohort (Supplementary Table 3).

Sensitivity analyses of the numbers of events for each endpoint in this cohort are presented in Supplementary Table 4. Specifically, women showed a significantly higher incidence of the primary endpoint after multivariable Cox regression analysis, which was adjusted for age, CCI, income, and clinical variables including dyslipidemia, IHD requiring coronary intervention, chronic kidney disease, atrial fibrillation, and use of beta-blocker (HR 1.52, 95% CI 1.27–1.82; *p* < 0.001). This finding was also mainly influenced by the significantly higher incidence of new-onset HF admission (HR 1.66, 95% CI 1.37–2.01; *p* < 0.001). There was no significant difference in the incidences of cardiovascular death for both sexes (HR 1.31, 95% CI 0.90–1.89; *p* = 0.154). The incidence of all-cause death for women and men were also similar (*p* = 0.579).

## Discussion

We here investigated the sex-related differences in the prognosis of patients with HCM. To the best of our knowledge, we have included the largest ever number of patients with HCM with the aim of evaluating the sex-related differences in their prognosis. In this study, women with HCM were generally older, had more comorbidities, and lower socioeconomic status, but were less likely to have IHD requiring coronary intervention and a history of myocardial infarction or chronic kidney disease than men with HCM. In the original cohort, the PS-matched cohort, and the patients without hypertension cohort (i.e., the cohort for sensitivity analysis), women with HCM consistently had poorer prognosis for the primary endpoint than men. This outcome was mainly due to a higher incidence of new-onset HF admission among women. However, there was no difference in the incidence of cardiovascular or all-cause death between the sexes.

For the last two decades, several studies were conducted on patients from different groups and of different ethnicities to determine whether women with HCM have poorer prognosis than men with HCM^3,6,7,13,15,17–20^. In a recent study by Geske et al., which had a large sample size and the longest follow-up duration so far, women with HCM had a significantly higher incidence of all-cause mortality than both men with HCM and the general population^6^. However, two other large studies by Olivotto et al. and Rowin et al. did not show any difference in survival rate between the sexes; the mean ages of the patients in the latter two studies were lower than that in the study by Geske et al. (mean age 42 and 46 years vs. 55)^3,6,7^. Our study’s mean age of the patients was 51, which may explain the insignificant difference in survival rate between the sexes. There are some plausible reasons why there was no sex-related difference in mortality rates. First, we focused on the association of sex with clinical course and survival in HCM. The age of patients in the original cohort was slightly younger, and thus the incidence of cardiovascular or all-cause death was so lower than that of the elderly general population that there may be no statistical difference between the two groups. Second, although the incidence of HF was higher among women, the incidence of HF-related death, one of the main causes of HCM-related death, might have decreased recently^7^. In a previous study of the HCM population in Korea, the proportion of implantable cardioverter defibrillator (ICD) implanted for primary prevention purposes was similar to that of large HCM cohorts of other countries (2.9% each)^7,21^. Since the number of patients with HCM in our study was greater than those of other previous studies, we could more actively adjust for the older age of women with HCM or referral bias using several statistical methods. Nevertheless, a well-designed prospective cohort study of patients of different ethnicities will be needed to determine the association of sex with the prognosis of HCM patients.

As reported in previous studies, our data showed a higher incidence of new-onset HF admission among women with HCM than among men^7,22^. One of the possible reasons for this might be the differences in cardiac chamber sizes between sexes. Investigations conducted using transthoracic echocardiography^23^ and cardiac magnetic resonance imaging before and after correction for body surface area showed that both women in the healthy population^24,25^ and HCM mutation carriers^20^ have smaller left ventricular (LV) end-diastolic dimensions and volumes than men. Nevertheless, there is no sex-related difference in the diagnostic criterion for HCM, i.e., a maximal LV wall thickness of at least 15 mm (or 13 mm in first-degree relatives)^26^. If the LV wall thickness of women reached a cutoff value enough to elicit a diagnosis of HCM, the LV dimensions and volumes in women with HCM would be relatively smaller than that of men. This may be the cause of the higher peak LV outflow gradient at initial diagnosis recorded in previous studies^6,7,22^ and higher incidence of new-onset HF admission in the present study. Second, women have a higher prevalence of HF with preserved ejection fraction (HFpEF) than men in the general population due to lower diastolic reserve, more frequent age-associated ventricular-arterial uncoupling, and smaller vessel size^27,28^. This trend was also observed in the HCM cohort; women with HCM had higher early mitral inflow velocity/early mitral annular velocity ratio and elevated right ventricular systolic pressure, both of which are parameters used for estimating LV filling pressure^6,18^. The beneficial effect of beta-blocker in patients with HCM was noted in a pre-specified subgroup analysis (Supplementary Fig. 2), which may be mediated by decreased inotropy with a decreased LV outflow pressure gradient and reduced heart rate with increased time for diastolic filling^6,29^. Third, in the present study, a higher proportion of women had income below the lowest 20% than men. In a previous study, the socioeconomic status affected the incidence and long-term outcomes of patients with HF^30^. Another international multicenter study also showed that women had higher Gini coefficients, which means more income inequality and can result in worse HF outcomes, even though they had fewer comorbidities than men^31^. Besides, decreased estrogen levels after menopause can partially explain the possible mechanisms underlying the poor HF outcomes in women with HCM. Specifically, LV mass in female mice with LV hypertrophy has been reported to increase after ovariectomy^32^. In another animal study, estrogen compounds had a proapoptotic effect and were not protective in the setting of HCM^33^. Although the lack of echocardiographic and biological data in the NHIS database makes it difficult to demonstrate the mechanisms underlying the poor HF outcome among the women with HCM in the present study, our findings are concordant with those of the previous studies^7,22^. The HR of the largest sample size in the present study also had a greater magnitude than those of previous studies after adjustment with several statistical methods.

Our study has some limitations. First, since this was a nationwide cohort study conducted using the NHIS database, we could not evaluate the echocardiographic parameters, such as LV peak pressure gradient, or cardiac magnetic resonance imaging results of the patients enrolled. This is the inherent limitation of national claims data. Additionally, we could not analyze the morphological phenotype of HCM or the prevalence of obstructive HCM, either. However, the diagnosis of HCM in Korea is strictly controlled periodically by the NHIS and a medical expert from the Health Insurance Review and Assessment Service (HIRA)^34^. Further, the ICD-10 and Rare Intractable Disease (RID) code-based diagnosis of HCM was well-matched with a previous echocardiography-based diagnosis of HCM^35^. Second, a previous study suggested that women with HCM had a smaller LV cavity size than men and that elderly women with HCM had a higher peak LV outflow pressure gradient due to gender-dependent contractile force and different structural remodeling^36^. Annual trends in number of diagnoses and age at diagnosis are both increasing, which seems to be irrespective of geographical or ethnicity^5,37^. This phenomenon might be due to increased physician awareness, diagnostic sensitivity or unknown mechanism related with sex-difference according to age. The present study only enrolled patients aged < 65 years, and thus we cannot investigate gender-specific changes in LV systolic function and remodeling pattern with age. However, exclusion of older HCM patients may be reasonable because LV wall thickness tends to increase in older age due to hidden hypertension or unknown causes^38^. We could focus on the association of sex with prognosis in patients with HCM by enrolling relatively young patients. Third, the prevalence of hypertension, which complicates a strict diagnosis of HCM, looks higher. However, the proportion of patients with hypertension in the present study was similar to that in other previous studies of HCM cohorts^6,7^. Furthermore, the results in the original cohort were concordant with those in the HCM patients without hypertension of the sensitivity analysis.

In conclusion, women with HCM have a higher incidence of the primary endpoint than men with HCM. This result was mainly driven by a higher new-onset HF admission in women with no difference in all-cause or cardiovascular mortality between women and men.

## Methods

### Data source and study population

**Figure 3:**
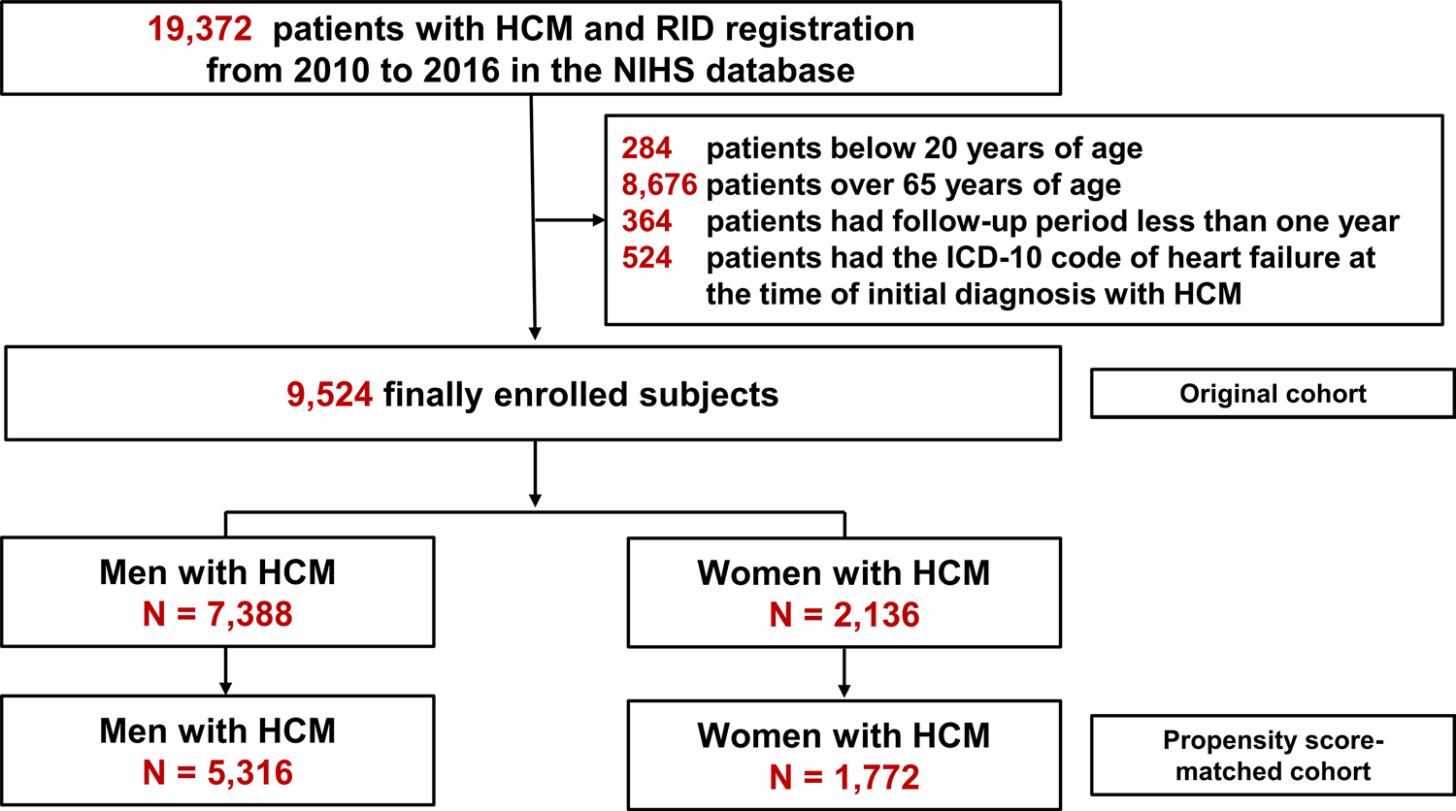
Flow chart of the study participants. *HCM* hypertrophic cardiomyopathy, *RID* rare intractable disease.

We conducted this nationwide population-based cohort study using the Korean NHIS claims database. The Korean NHIS is a mandatory universal health insurance program managed by the Korean government since 1989 and offers comprehensive medical care to 97% of the Korean population^39^. The remaining 3% of Koreans with evidence of low income are covered by the Medical Aid Program; information from this program has been incorporated into a single database since 2006. The NHIS database includes detailed information of each registered individual, including data on demographic characteristics, health behavior, diagnoses, prescriptions, surgeries or procedures, and health care utilization (i.e., hospitalization)^39^. This database has been previously used in many published studies, and its validity as a reliable data source has been established^40–42^. Additionally, the HIRA system provides regular evaluation data, quality control data, and feedback about the whole medical care system in Korea. Through the cooperation of the NHIS and HIRA, a large database of the medical information of all Koreans was established under the strict supervision of the Ministry of Health and Welfare^34^. This database encompasses information on demographics, medical facility utilization history, diagnoses, prescriptions, and the national health exam results of a given year^43^. From this database, we selected subjects who were aged ≥ 20 years between January 1, 2010, and December 31, 2016. To minimize the possibility of hypertrophic changes of the myocardium in older people^38^, 8676 patients aged > 65 years were excluded. We also excluded 364 patients who had less than a year of follow-up and 524 patients who had the ICD-10 code of HF in their record at the time of enrollment. A total of 9524 patients with HCM were finally included in the study (Fig. 3). This study complied with the provisions of the 2013 Declaration of Helsinki. The study was approved by the Institutional Review Board of Seoul National University Hospital (E-1092-058-1009), and the need for informed consent was waived by the Institutional Review Board of Seoul National University Hospital due to the retrospective nature of the study.

### Diagnosis of HCM and its validity

HCM was defined as (1) record of at least one admission or outpatient clinic visit with ICD-10 code of I42.1 or I42.2; and (2) registration in the RID program (code of V127). In Korea, HCM falls under the RID category, in which patients are designated as special medical aid beneficiaries with expanding benefit of the NHIS. Since 2006, the Korean government has introduced an initiative covering 90% of all medical expenses claimed by these patients. Therefore, the diagnosis of HCM is strictly determined and monitored via thorough verification using clinical and imaging evidences, and periodical reviews by medical experts and health insurance professionals, according to an act established by the Ministry of Health and Welfare. Furthermore, the definition of HCM according to a diagnostic code was validated by our institution in a previous study, by reviewing medical records, including echocardiography or cardiac magnetic resonance imaging data, and comparing the diagnostic accuracy of the code^35^.

### Covariates

Previously published detailed methods were adopted for this study^35,44,45^. Age and sex data were retrieved using resident identification numbers. Income level was dichotomized at the lowest 20% and was presented as a categorical variable. The detailed definition of comorbidities, including hypertension, diabetes mellitus, dyslipidemia, IHD (requiring stent insertion), myocardial infarction, peripheral artery disease, atrial fibrillation, ischemic/bleeding stroke, chronic kidney disease / end-stage renal disease is summarized in Supplementary Table 5. CCI was also calculated as previously described^46,47^. Prescription lists, including renin–angiotensin–aldosterone system blocker, beta-blocker, calcium channel blocker, statin, and antiplatelet agent, were ascertained.

### Study outcome and follow-up

The primary endpoint was defined as a composite of cardiovascular death and new-onset HF. The secondary endpoint included the individual components of the primary endpoint and all-cause death. New-onset HF was defined as a newly acquired I50 diagnostic code on admission after the diagnostic code for HCM was established. The study subjects were followed up until the occurrence of each endpoint or until December 31, 2017, whichever came first.

### Statistical analysis

Descriptive statistics were presented as means ± standard deviations or medians (IQRs) for continuous variables and numbers (percentages) for categorical variables. For the comparison between groups, the unpaired Student’s *t*-test was applied for continuous variables, and the χ^2^ test or Fisher’s exact test was used for categorical variables, as appropriate. The incidence rates of death or new-onset HF events were calculated by dividing the number of detected cases by the follow-up duration, and were presented as a value per 1000 person-years. Kaplan–Meier curves were used to present the cumulative incidence of the primary and secondary endpoints by using a log-rank test for statistical analysis. Multivariable Cox proportional-hazards analysis with two nested models were used to identify the independent risk factors for the primary and secondary endpoints, which were expressed as HRs and corresponding 95% CIs. The variables with a *p*-value of < 0.10 for the primary endpoint in the univariable Cox analysis were selected and included in the multivariable Cox analysis.

To adjust for the imbalance between the baseline characteristics of men and women, we used two different approaches. First, the multivariable Cox regression analysis was performed while adjusting for the baseline characteristics that were significantly different in the univariable analysis. Second, the PS-matching analysis was adopted to balance the differences in covariates. It was performed with a 3:1 greedy matching technique. We set a caliper for nearest-neighbor matching within the first four to eight digits. For sensitivity analysis, we excluded 3,956 patients with ICD-10 codes indicating hypertension (I10-13, I15) in their records and repeated the statistical analyses.

All statistical analyses were executed using the SAS software, Version 9.4 of SAS System for Windows, Copyright 2021 (SAS Institute Inc., Cary, NC), and R version 3.6.0 software (R Development Core Team, Vienna, Austria). A two-sided *p*-value of < 0.05 was considered a significant difference.

## Supplementary Information

Supplementary Information.

## Data Availability

The data is unavailable outside NHIS system.
