# Supplementary material for: Sex differences in the prognosis of patients with hypertrophic cardiomyopathy
Source: Sci Rep. 2021 Mar 1;11:4854. doi: 10.1038/s41598-021-84335-1 (PMC7921653; doi:10.1038/s41598-021-84335-1)
Supplement: Supplementary file 1 — Supplementary Information. [file 41598_2021_84335_MOESM1_ESM.docx]

**Sex Differences in the Prognosis of Patients with Hypertrophic Cardiomyopathy**

**Running Title:** Sex differences and prognosis in HCM

Minkwan Kim, MD, PhD^1,2^, Bongsung Kim, PhD^3^, You-Jung Choi, MD, PhD^1,^

Hyun-Jung Lee, MD^1^, Heesun Lee, MD^4^, Jun-Bean Park, MD, PhD^1^, Seung-Pyo Lee, MD, PhD^1^, Kyung-Do Han, PhD^3^, Yong-Jin Kim, MD, PhD^1^, *Hyung-Kwan Kim, MD, PhD^1^

^1^Department of Internal Medicine and Cardiovascular Center, Seoul National University Hospital, Seoul, Republic of Korea

^2^Division of Cardiology, Department of Internal Medicine, Yongin Severance Hospital, Yonsei University College of Medicine, Yongin-si, Gyeonggi-do, Republic of Korea

^3^Department of Statistics and Actuarial Science, The Soongsil University, Seoul, Republic of Korea

^4^Division of Cardiology, Department of Internal Medicine, Seoul National University Hospital Healthcare System Gangnam Center, Seoul, Korea

**Supplementary materials**

**Contents**

**I. Supplementary Tables**

**II. Supplementary Figure**

**I. Supplementary Tables**

**Supplementary Table 1. Univariate Cox regression for the primary endpoint in the original cohort of patients with HCM.**

|  | **HR** | **95% CI** | ***p*** |
| --- | --- | --- | --- |
| Female sex | 1.49 | 1.30–1.71 | <0.001 |
| Age, per 1 year | 1.02 | 1.30–1.71 | <0.001 |
| Income, lower 20% | 1.43 | 1.23–1.67 | <0.001 |
| Hypertension | 1.55 | 1.35–1.77 | <0.001 |
| Dyslipidemia | 1.16 | 0.99–1.37 | 0.070 |
| Chronic kidney disease | 3.06 | 2.27–4.12 | <0.001 |
| IHD requiring coronary intervention | 1.99 | 1.15–3.44 | 0.014 |
| Atrial fibrillation | 2.71 | 2.22–3.32 | <0.001 |
| Beta-blocker | 1.44 | 1.25–1.64 | <0.001 |
| Charlson comorbidity index, per 1 point | 1.19 | 1.15–1.22 | <0.001 |

HCM, hypertrophic cardiomyopathy; HR, hazard ratio; CI, confidence interval; IHD, ischemic heart disease.

**Supplementary Table 2. Baseline clinical characteristics before and after propensity score matching**

|  | **Original cohort** | | | **Propensity score-matched cohort** | | |
| --- | --- | --- | --- | --- | --- | --- |
|  | **Men** | **Women** | **ASD** | **Men** | **Women** | **ASD** |
|  | **(n=7,388)** | **(n=2,136)** |  | **(n=5,316)** | **(n=1,772)** |  |
| Age | 51.4 ± 9.1 | 52.6 ± 9.7 | 0.128 | 51.7 ± 9.2 | 51.9 ± 10.1 | 0.022 |
| Income, low 20% | 1133 (15.3) | 483 (22.6) | 0.186 | 728 (13.7) | 249 (14.1) | 0.010 |
| *Underlying disease, n (%)* |  |  |  |  |  |  |
| Hypertension | 703 (9.5) | 187 (8.8) | 0.026 | 508 (9.56) | 149 (8.4) | 0.040 |
| Diabetes mellitus | 3089 (41.8) | 867 (40.6) | 0.025 | 2215 (41.7) | 734 (41.4) | 0.005 |
| Dyslipidemia | 1819 (24.6) | 527 (24.7) | 0.001 | 1290 (24.3) | 423 (23.9) | 0.009 |
| IHD requiring coronary intervention | 82 (1.1) | 12 (0.6) | 0.060 | 30 (0.6) | 12 (0.7) | 0.014 |
| Myocardial infarction | 203 (2.8) | 40 (1.9) | 0.058 | 131 (2.5) | 27 (1.5) | 0.067 |
| Peripheral artery disease | 488 (6.6) | 189 (8.9) | 0.084 | 357 (6.7) | 145 (8.2) | 0.056 |
| Atrial fibrillation | 479 (6.5) | 133 (6.2) | 0.011 | 331 (6.2) | 121 (6.8) | 0.024 |
| Previous stroke | 323 (4.4) | 97 (4.5) | 0.008 | 235 (4.4) | 64 (3.6) | 0.041 |
| Chronic kidney disease | 195 (2.6) | 39 (1.8) | 0.055 | 120 (2.3) | 36 (2.0) | 0.016 |
| End-stage renal disease | 51 (0.7) | 12 (0.6) | 0.016 | 35 (0.7) | 10 (0.6) | 0.012 |
| *Current medication, n (%)* |  |  |  |  |  |  |
| RAS blocker | 2307 (31.2) | 627 (29.4) | 0.041 | 1669 (31.4) | 526 (29.7) | 0.037 |
| Beta-blocker | 2628 (35.6) | 759 (35.5) | 0.001 | 1835 (34.5) | 617 (34.8) | 0.006 |
| Calcium channel blocker | 1087 (14.7) | 306 (14.3) | 0.011 | 767 (14.4) | 249 (14.1) | 0.011 |
| Antiplatelet agent | 2188 (29.6) | 595 (27.9) | 0.039 | 1525 (28.7) | 483 (27.3) | 0.032 |
| Statin | 1803 (24.4) | 531 (24.9) | 0.011 | 1272 (23.9) | 420 (23.7) | 0.005 |
| Charlson comorbidity index | 1.95 ± 1.89 | 2.31 ± 1.98 | 0.186 | 1.97 ± 1.81 | 2.00 ± 1.77 | 0.016 |

ASD, absolute standardized difference; IHD, ischemic heart disease; RAS, renin-angiotensin system

**Supplementary Table 3. Baseline clinical characteristics in HCM patients without hypertension.**

|  | **Total**  **(N=5,568)** | **Men  (N=4,299)** | **Women  (N=1,269)** | ***p*** |
| --- | --- | --- | --- | --- |
| Age | 50.6 ± 9.7 | 50.4 ± 9.5 | 51.2 ± 10.4 | 0.016 |
| Income, low 20% | 847 (15.2) | 592 (13.8) | 255 (20.1) | <0.001 |
| *Underlying disease, n (%)* |  |  |  |  |
| Diabetes mellitus | 212 (3.8) | 168 (3.9) | 44 (3.5) | 0.471 |
| Dyslipidemia | 557 (10.0) | 435 (10.1) | 122 (9.6) | 0.599 |
| IHD requiring coronary intervention | 20 (0.4) | 19 (0.4) | 1 (0.1) | 0.057 |
| Myocardial infarction | 70 (1.3) | 58 (1.4) | 12 (1.0) | 0.257 |
| Peripheral artery disease | 168 (3.0) | 121 (2.8) | 47 (3.7) | 0.104 |
| Atrial fibrillation | 159 (2.9) | 121 (2.8) | 38 (3.0) | 0.735 |
| Previous stroke | 85 (1.5) | 60 (1.4) | 25 (2.0) | 0.143 |
| Chronic kidney disease | 45 (0.8) | 38 (0.9) | 7 (0.6) | 0.245 |
| End-stage renal disease | 11 (0.2) | 9 (0.2) | 2 (0.2) | 0.715 |
| *Current medication, n (%)* |  |  |  |  |
| RAS blocker | 163 (2.9) | 126 (2.9) | 37 (2.9) | 0.977 |
| Beta-blocker | 718 (12.9) | 549 (12.77) | 169 (13.32) | 0.609 |
| Calcium channel blocker | 62 (1.11) | 52 (1.21) | 10 (0.79) | 0.209 |
| Antiplatelet agent | 604 (10.9) | 482 (11.2) | 122 (9.6) | 0.108 |
| Statin | 566 (10.2) | 442 (10.3) | 124 (9.8) | 0.597 |
| Charlson comorbidity index | 1.66 ± 1.70 | 1.57 ± 1.67 | 1.96 ± 1.77 | <0.001 |

HCM, hypertrophic cardiomyopathy; IHD, ischemic heart disease; RAS, renin-angiotensin system

**Supplementary Table 4. Sex difference in the Cox regression analysis in the sensitivity analysis of HCM patients without hypertension.**

|  | **Number** | **Event** | **IR (per 1,000)** | **Unadjusted** | ***p*** | **Model 1*** | ***p*** | **Model 2†** | ***p*** |
| --- | --- | --- | --- | --- | --- | --- | --- | --- | --- |
| **Primary endpoint** | |  |  |  |  |  |  |  |  |
| Men (n=4,299) | 4,299 | 384 | 19.87 | 1 (reference) |  | 1 (reference) |  | 1 (reference) |  |
| Women (n=1,269) | 1,269 | 181 | 32.04 | 1.60 (1.34–1.91) | <0.001 | 1.52 (1.27–1.81) | <0.001 | 1.52 (1.27–1.82) | <0.001 |
| **Cardiovascular death** |  |  |  |  |  |  |  |  |  |
| Men (n=4,299) | 4299 | 96 | 4.81 | 1 (reference) |  | 1 (reference) |  | 1 (reference) |  |
| Women (n=1,269) | 1,269 | 41 | 6.79 | 1.37 (0.95–1.98) | 0.091 | 1.27 (0.875, 1.827) | 0.211 | 1.31 (0.90–1.89) | 0.154 |
| **New-onset HF** |  |  |  |  |  |  |  |  |  |
| Men (n=4,299) | 4,299 | 317 | 16.41 | 1 (reference) |  | 1 (reference) |  | 1 (reference) |  |
| Women (n=1,269) | 1,269 | 163 | 28.85 | 1.76 (1.45–2.12) | <0.001 | 1.66 (1.38–2.01) | <0.001 | 1.66 (1.37–2.01) | <0.001 |
| **All-cause Death** |  |  |  |  |  |  |  |  |  |
| Men (n=4,299) | 4,299 | 178 | 8.91 | 1 (reference) |  | 1 (reference) |  | 1 (reference) |  |
| Women (n=1,269) | 1,269 | 55 | 9.11 | 1.02 (0.75–1.38) | 0.907 | 0.92 (0.68–1.24) | 0.573 | 0.92 (0.68–1.25) | 0.579 |

* Multivariate clinical model 1 was adjusted for age and Charlson comorbidity index (CCI). **†** Model 2 was adjusted for age, CCI, income, dyslipidemia, chronic kidney disease, ischemic heart disease requiring coronary intervention, atrial fibrillation, and beta-blocker. HCM, hypertrophic cardiomyopathy; IR, incident rate; HF, heart failure

**Supplementary Table 5. Definitions of covariates.**

| **Diagnosis** | **ICD-10-CM code and definition** | **Diagnostic definition** |
| --- | --- | --- |
| **Hypertension** | I10-I13, I15; and minimum 1 prescription of anti-hypertensive drug (thiazide, loop diuretics, aldosterone antagonist, alpha-/beta-blocker, calcium channel blocker, renin-angiotensin system blocker). | Admission≥1 or outpatient department≥2 |
| **Diabetes mellitus** | E11-E14; and minimum 1 prescription of anti-diabetic drugs (sulfonylureas, metformin, meglitinides, thiazolidinediones, dipeptidyl peptidase-4 inhibitors, α-glucosidase inhibitors, and SGLT2-inhibitor, GLP-1 agonist, or insulin). | Admission≥1 or outpatient department≥2 |
| **Dyslipidemia** | E78, and minimum 1 prescription of lipid-lowering medication (statin, ezetimibe, fenofibrate) | Admission≥1 or outpatient department≥2 |
| **Ischemic heart disease requiring coronary intervention** | I20, I23-25; and procedure code of M6561-6567 | Admission ≥ 1 or outpatient clinic ≥ 2 |
| **Myocardial infarction** | I21, I22 | Admission ≥ 1 or outpatient clinic ≥ 2 |
| **Peripheral artery disease** | I70.2, I73 | Admission≥1 or outpatient clinic ≥ 2 |
| **Heart failure** | I50 | Admission≥1 or outpatient clinic ≥ 2 |
| **Atrial fibrillation** | I48 | Admission≥1 or outpatient clinic ≥ 2 |
| **Previous stroke** | I63, I64 | Admission≥1 or outpatient clinic ≥ 2 |
| **Chronic kidney disease** | N00-007, N11, I12, N18-19, Q61 | Admission≥1 or outpatient clinic ≥ 2 |
| **End-stage renal disease** | N18.5, N18.9, N19, Z49, Z99.2; or (Procedure codes) O7011-7020 (Hemodialysis), O7071-O7075 (Peritoneal dialysis); or V001(Hemodialysis), V003 (Peritoneal dialysis) | Dialysis ≥ 2 |

**II. Supplementary Figure**

**Supplementary Figure 1. Kaplan-Meier curve of the cumulative incidence of clinical events in the propensity score-matched cohort of patients with hypertrophic cardiomyopathy.**

**
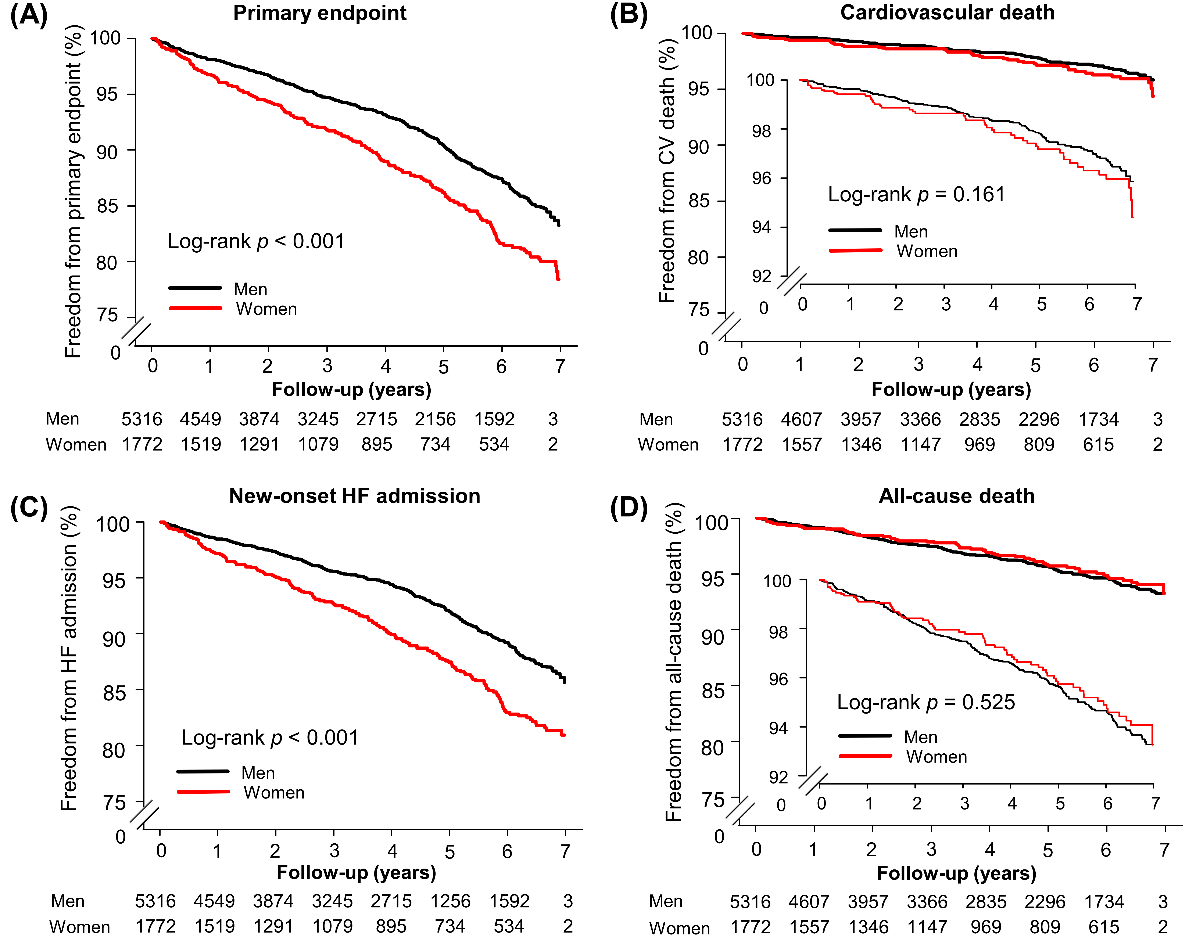
**(A) the composite endpoints of cardiovascular death or admission due to new-onset heart failure, (B) Cardiovascular death, (C) admission due to new-onset heart failure, (D) all-cause death.

CV, cardiovascular; HF, heart failure.

**Supplementary Figure 2. Adjusted hazard ratio (HR) of variables to predict prognosis in women with hypertrophic cardiomyopathy (HCM).**

Women with HCM had poor prognosis regardless of age, atrial fibrillation, hypertension, stroke, chronic kidney disease, CCI over 2 points, which means more comorbidities. Note that women with HCM taking beta-blocker had a lower adjusted HR, which means lowering the risk from the future composite event than without.


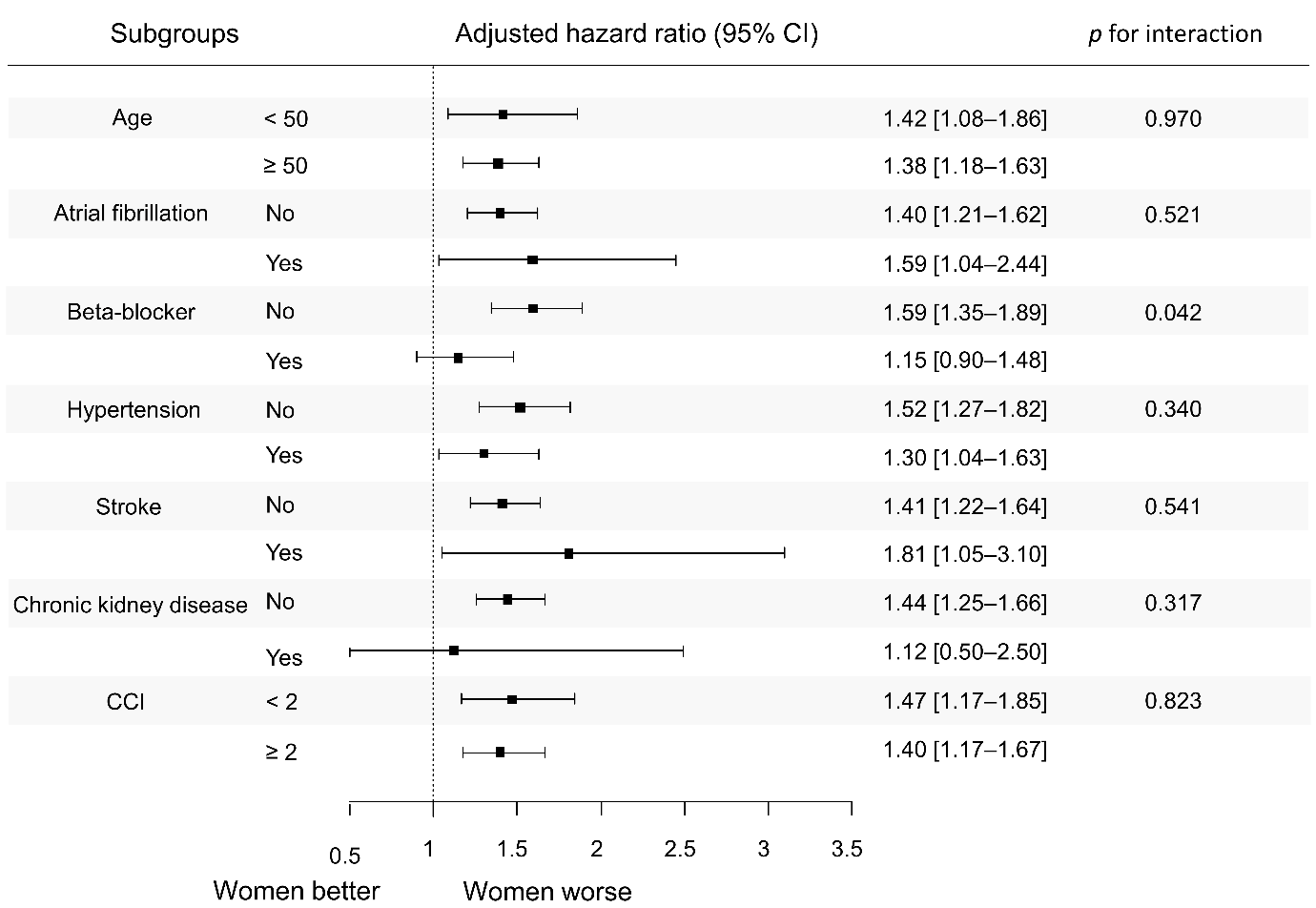


CCI, Charlson comorbidity index.
